# Diversity of biting midges, mosquitoes and sand flies at four dog shelters in rural and peri-urban areas of Central Morocco[Fn FN1]

**DOI:** 10.1051/parasite/2024057

**Published:** 2024-09-27

**Authors:** Abderrahmane Zahri, Mehdi Ahlamine, Fatima-Zahra Abou-Elaaz, Hasnaa Talimi, Ikhlass El Berbri, Thomas Balenghien, Maria Bourquia

**Affiliations:** 1 Parasitology and Parasitic Diseases Unit, Department of Animal Pathology and Public Health, Institut Agronomique et Vétérinaire Hassan II Rabat Morocco; 2 Geophysics, Natural Patrimony and Green Chemistry Research Centre (GEOPAC), Geo-Biodiversity and Natural Patrimony Laboratory (GEOBIOL), Scientific Institute, Mohammed V University Rabat Morocco; 3 Laboratory of Parasitology and Vector-Borne-Diseases, Institut Pasteur du Maroc Casablanca Morocco; 4 Systems and Data Engineering Team, National School of Applied Sciences, Abdelmalek Essaâdi University Tangier Morocco; 5 Microbiology, Immunology and Contagious Diseases Unit, Department of Animal Pathology and Public Health, Institut Agronomique et Vétérinaire Hassan II Rabat Morocco; 6 CIRAD, UMR ASTRE 34398 Montpellier France; 7 ASTRE, Université de Montpellier, CIRAD, INRAE Montpellier France

**Keywords:** Diversity, Biting midges, Mosquitoes, Sand flies, Dog shelters, Morocco

## Abstract

Blood-feeding arthropods are involved in the transmission of several pathogens that have a major impact on public health. Entomological investigations highlighted the composition, abundance, and diversity of flying hematophagous arthropods at four dog shelters located in central Morocco during an eight-month study, with the aim of discussing their vectorial roles and assessing the risk of these shelters as foci for zoonotic diseases. Monitoring of the arthropod fauna for 64 catch nights resulted in the collection of 2,321 biting midges (Ceratopogonidae), 570 mosquitoes (Culicidae), and 475 sand flies (Psychodidae). Fourteen *Culicoides* species were recorded and dominant species were *Culicoides imicola* (55.96%), *C. paolae* (16.07%), *C. circumscriptus* (10.29%), and *C. newsteadi* (5.77%). Three mosquito species were collected, including *Culex pipiens* s.l. (96.84%), *Culiseta longiareolata* (2.80%), and *Cx. perexiguus* (0.36%). Ten sand fly species were collected, including seven *Phlebotomus* species (62.70%) and three *Sergentomyia* species (37.30%); *Sergentomyia minuta* was the most dominant species (34.31%), followed by *Phlebotomus sergenti* (32.42%), typical *Ph. perniciosus* (8.63%), *Ph. alexandri* (6.94%), and *Ph. riouxi* (6.52%). The coexistence of several vectors in these study areas indicates the potential circulation of a wide range of pathogens, including zoonotic ones, thus requiring the implementation of surveillance and control programs to prevent the emergence and spread of disease outbreaks.

## Abstract (fr)

Les arthropodes hématophages sont impliqués dans la transmission de plusieurs agents pathogènes ayant un impact majeur sur la santé publique. Des investigations entomologiques ont mis en évidence la composition, l’abondance et la diversité des arthropodes hématophages volants dans quatre chenils situés au centre du Maroc au cours d’une étude de huit mois, dans le but de discuter de leurs rôles vectoriels et d’évaluer le risque de ces chenils comme foyers de maladies zoonotiques. Le suivi de la faune des arthropodes au cours de 64 nuits de capture a permis la collecte de 2 321 culicoïdes (Ceratopogonidae), 570 moustiques (Culicidae) et 475 phlébotomes (Psychodidae). Quatorze espèces de *Culicoides* ont été recensées et les espèces dominantes étaient *Culicoides imicola* (55,96 %), *C. paolae* (16,07 %), *C. circumscriptus* (10,29 %) et *C. newsteadi* (5,77 %). Trois espèces de moustiques ont été collectées, dont *Culex pipiens s.l.* (96,84 %), *Culiseta longiareolata* (2,80 %) et *Cx. perexiguus* (0,36 %). Dix espèces de phlébotomes ont été collectées, dont sept espèces de *Phlebotomus* (62,70 %) et trois espèces de *Sergentomyia* (37,30 %); *Sergentomyia minuta* était l’espèce dominante (34,31 %), suivie de *Phlebotomus sergenti* (32,42 %), *Ph. perniciosus* typique (8,63 %), *Ph. alexandri* (6,94 %) et *Ph. riouxi* (6,52 %). La coexistence de plusieurs vecteurs dans ces zones d’étude indique la circulation potentielle d’un large éventail d’agents pathogènes, y compris zoonotiques, ce qui nécessite la mise en œuvre de programmes de surveillance et de contrôle pour prévenir l’émergence et la propagation d’épidémies.

## Introduction

Blood-sucking arthropods, such as ticks, fleas, mosquitoes and phlebotomine sand flies, are responsible for the transmission of a large number of pathogens including bacteria, viruses, protozoa, and helminths, which can have a significant impact on human and animal health, and on the global economy through considerable economic losses [61, 84]. The global spread of these vectors and their transmitted pathogens is driven by many factors, mainly related to climatic and environmental changes, the evolution of international trade, the movement of human and animal populations, or resistance developed to insecticidal compounds used for insect control [62, 84]. As a consequence, several human outbreaks of vector-borne diseases have recently been reported on different continents. In Africa, the last confirmed outbreak of dengue fever, a mosquito-borne flavivirus, was described in Tanzania in 2019 [48]. Furthermore, the largest epidemics ever seen of West Nile fever, caused by a virus transmitted by the bites of *Culex* mosquitoes, was observed in the United States of America between 2002 and 2003 [79], whereas outbreaks regularly occurred in several central European countries in 2018, demonstrating intense transmission [23]. Moreover, two major outbreaks of visceral leishmaniasis due to *Leishmania donovani* transmitted by *Phlebotomus argentipes* sand flies were documented in the Indian sub-continent in 2014 and 2018 [64, 83]. The south of Venezuela and north of Brazil experienced massive outbreaks of Amazonian onchocerciasis (river blindness), which is a neglected tropical disease transmitted by simuliid black flies [31]. So far, the impact of biting midges (Diptera: Ceratopogonidae) on public health is limited to the transmission of Oropouche Fever Virus (ORFV), which causes a febrile illness in humans in Central and South America [26, 96], and leading currently to health impacts in French Guiana [75]. However, they are competent vectors for other viruses of veterinary interest, such as Schmallenberg Virus (SBV), Bluetongue Virus (BTV) affecting livestock [9, 63], African Horse Sickness Virus (AHSV), which causes a fatal disease in the equine population [27], and Epizootic Hemorrhagic Disease Virus (EHDV), which is booming in Europe [93].

Dogs are reservoir hosts of many vector-borne zoonotic pathogens (*e.g.*, *Anaplasma phagocytophilum* and *A. platys, Borrelia burgdorferi* sensu lato complex, *Ehrlichia canis* and *E. ewingii, Dirofilaria immitis* and *D. repens, Leishmania infantum, Rickettsia conorii* and *R. rickettsii, Dipylidium caninum, Babesia canis vogeli* and *B. canis rossi*) [84], most of which has previously been reported in Morocco [12, 39, 40, 88, 94, 95]. Dog populations have increased remarkably in developed and developing countries, with dogs playing a major role in supporting people with mental illnesses and physical disabilities [81]. At the same time, the number of stray dogs has increased in developing countries due to ineffective control programs [57]. They are often confined to shelters which are located in rural and peri-urban areas and are exposed to the risk of infection by various vector-borne pathogens [57]. In addition, they are suffering from a lack of the necessary veterinary care and adequate preventive measures [57]. Therefore, they represent high-risk foci for workers and visitors, who may be exposed to zoonotic pathogens [51]. Although the diversity of sand flies, mosquitoes, and *Culicoides* has been studied in Morocco [3, 15, 101, 104], knowledge of arthropod fauna in shelters, which is essential for assessing the risk of transmission of vector-borne zoonotic pathogens, remains undescribed.

Thus, the aim of this work was to (a) describe the populations of biting midges, mosquitoes, and sand flies in four dog shelters located in central Morocco, (b) discuss their potential involvement as vectors in the transmission of various pathogens, and (c) assess the risk of these shelters as foci for zoonotic diseases.

## Material and methods

### Study area

This study was carried out in four dog shelters (DS) located in four different regions (DS1, Rabat-Salé-Kénitra region; DS2, Casablanca-Settat region; DS3, Fez-Meknès region and DS4, Béni Mellal-Khénifra region) of central Morocco. These regions are characterized by a Mediterranean climate with mild, wet winters and hot, dry summers. DS1 (33.806538, −6.902472) is located in the rural district of Sidi Yahya Zaër, while the others are in peri-urban areas at the city edges of Bouskoura (DS2, 33.507278, −7.631269), Fez (DS3, 34.004291, −5.106422), and Khouribga (DS4, 32.845631, −6.938536). DS1, DS2, and DS4 are constituted by large, multiple and open dog kennels (3–5 dogs per kennel), whereas DS3 is characterized by several uncovered rooms, where dogs can roam freely. Additionally, there is a special room for abandoned cats in DS2 and DS3, and stalls for donkeys and mules without owners in DS2. All these shelters are well fenced. DS1 and DS2 are surrounded by forests and field crops, and DS3 and DS4 by a few green spaces.

### Insect collections and identification

 A single Onderstepoort Veterinary Institute (OVI) black light suction trap was placed at each DS to collect adult *Culicoides.* This 220 V trap was equipped with a 30 cm fluorescent UV neon tube (8 W) attracting insects and with a fan propelling them to a collection pot, filled with a buffer solution, which is used to limit nucleic acid degradation and consequently increase the probability of viral genome detection (RNAlater-like solution was prepared as described by Camacho-Sanchez *et al.* [22] using the following stock solutions and reagents: 0.5 M ethylenediaminetetraacetic acid (EDTA) disodium salt dihydrate (18.61 g/100 mL, pH to 8.0 with NaOH through stirring), 1 M sodium citrate salt dihydrate (29.4 g/100 mL, stirring used for dissolving), ammonium sulfate (powdered) and sterile water (see Appendix for the protocol used to prepare this solution). Two drops of commercial detergent (manufactured by Ecolab Maroc, Casablanca, Morocco) were added to the collection pot to help the drowning of *Culicoides*, avoiding their drying which complicates morphological identification.

Adult mosquitoes were collected using a BG-Pro mosquito trap (BGP), baited only with CO_2_ (without BG-Lure), installed at each DS, and hung at a tree branch where there is no access for dogs. The trap is powered by an external 6 V battery and equipped with white light bulbs, a fan, and a collector net. CO_2_ was produced with a fire extinguisher at a flow rate of 0.5 kg/day. The collected mosquitoes were transported live to the laboratory and then killed by freezing at −20 °C.

Sand flies were sampled using castor oil on sticky papers (20 × 20 cm) disposed on walls inside the different DS. 30 sticky papers were used on each trapping night in the DS concerned.

Trapping was carried out from April 1 to November 30, 2022 with two successive sampling nights per month and DS. The traps and sticky papers were functional from 6 pm to 10 am. The collected insects were sent directly to the Parasitology and parasitic diseases laboratory of the Institut Agronomique et Vétérinaire (IAV) Hassan II for morphological identification to the species level using relevant keys [7, 8, 35, 73]. These blood-feeding arthropods were stored at −80 °C.

### Data analysis

To explore the diversity of the collected insects, different parameters and ecological indexes were calculated using Microsoft Office Excel 2019 (Microsoft Corporation, Redmond, WA, USA): (i) the absolute abundance (N) (total number of specimens collected by the various traps used); (ii) the relative abundance (RA) [(the number of specimens of each species/the total number of specimens of the family) × 100]; (iii) the sex ratio (SR) [(the result of dividing the number of females by the number of males belonging to the same species collected for each family)]; (iv) the frequency of occurrence per DS (C (%)/DS) [(number of collections containing the species under consideration / total number of surveys carried out) × 100]; (v) the species richness (S) (number of all species collected for each family in each study area); and (vi) the Shannon and Wiener diversity index (H’)[(H’ = Σ Pi log2 Pi) and Pi is the proportion of all samples belonging to the “*i*-th” species]. Graphs were created using R version 4.2.0 with the ggplot2 R package [89].

## Results

### Species composition

During the 64 collection nights, a total of 2,321 *Culicoides* biting midges (Diptera: Ceratopogonidae) were collected, representing fourteen species with the dominance of *Culicoides imicola* (55.96%), followed by *C. paolae* (16.07%), *C. circumscriptus* (10.29%), and *C. newsteadi* (5.77%). The number of collected females was higher compared to males (1,723♀/598♂). The sex ratio differs depending on the species. Thirty-four engorged females were identified, dominated by *Culicoides imicola* (27/34) (Table 1).

**Table 1:** Total number, global abundance, rank, sex ratio, and occurrence per dog shelter (C (%)/DS) of collected biting midges (Ceratopogonidae: *Culicoides*) by UV-light/suction trap (OVI type), from April to November 2022, at four dog shelters (DS) in central Morocco.

Species	*T*	♀ (engorged females)	♂	Rank	Sex ratio	C (%)/DS	Percentage	Cumulative percentage
*C. imicola*	1,299	1,197 (27)	102	1	11.73	75	55.967	55.967
*C. paolae*	373	167	206	2	0.81	50	16.071	72.038
*C. circumscriptus*	239	103 (1)	136	3	0.75	50	10.297	82.335
*C. newsteadi*	134	120 (3)	14	4	8.57	75	5.773	88.108
*C. sahariensis*	51	14	37	5	0.37	50	2.197	90.305
*C. kingi*	50	20	30	6	0.66	50	2.154	92.459
*C. puncticollis*	42	26	16	7	1.62	50	1.810	94.269
*C. kurensis*	36	9	27	8	0.33	50	1.551	95.820
*C. saevus/langeroni*	35	24 (2)	11	9	2.18	50	1.508	97.328
*C. univittatus*	29	16	13	10	1.23	50	1.249	98.577
*C. longipennis*	12	10 (1)	2	11	5.00	25	0.517	99.094
*C. obsoletus/scoticus*	9	5	4	12	1.25	75	0.388	99.482
*C. submaritimus*	2	2	0	13	–	50	0.086	99.568
*C. clastrieri*	1	1	0	14	–	25	0.044	99.612
Damaged	9	9	0	–	–	50	0.388	–
Total	2,321	1,723 (34)	598	–	2.88	–	100	–

A total of 570 mosquito specimens (Diptera: Culicidae) were caught, belonging to two genera: *Culex* (97.20%) and *Culiseta* (2.80%) (Table 2). *Culex pipiens* s.l. was the most frequent species (96.84%) followed by *Culiseta longiareolata* (2.80%) and *Cx. perexiguus* (0.36%). Females were predominant *versus* males (525♀/45♂), which means they were favored in the sex ratio. Five of the 525 females were engorged.

**Table 2:** Total number, global abundance, rank, sex ratio, and occurrence per dog shelter (C (%)/DS) of caught mosquitoes (Culicidae) by BG-Pro mosquito trap (BGP), from April to November 2022, at four dog shelters (DS) in central Morocco.

Species	*T*	♀ (engorged females)	♂	Rank	Sex ratio	C (%)/DS	Percentage	Cumulative percentage
*Culex pipiens* s.l.	552	511 (5)	41	1	12.46	100	96.842	96.842
*Culiseta longiareolata*	16	12	4	2	3.00	100	2.807	99.649
*Culex perexiguus*	2	2	0	3	–	25	0.351	100
Total	570	525 (5)	45	–	11.66	–	100	–

A total of 475 sand flies (Diptera: Psychodidae) were recorded, belonging to two genera (Table 3). Seven *Phlebotomus* species (62.70%) and three *Sergentomyia* species (37.30%) were collected. *Sergentomyia minuta* was the most prevalent species (34.31%), followed by *Phlebotomus sergenti* (32.42%), typical *Ph. perniciosus* (PN) (8.63%), *Ph. alexandri* (6.94%), and *Ph. riouxi* (6.52%). The males outnumbered the females (133♀/342♂) and therefore, the sex ratio was favorable towards all male species except for *Sergentomyia fallax*. Twelve engorged females were found, with the dominance of *Phlebotomus sergenti* (10/12).

**Table 3:** Total number, global abundance, rank, sex ratio, and occurrence per dog shelter (C (%)/DS) of trapped sand flies (Psychodidae) by sticky traps, from April to November 2022, at four dog shelters (DS) in central Morocco. *Ph: Phlebotomus, Se: Sergentomyia.*

Species	*T*	♀ (engorged females)	♂	Rank	Sex ratio	C (%)/DS	Percentage	Cumulative percentage
*Se. minuta*	163	61 (2)	102	1	0.59	75	34.316	34.316
*Ph. sergenti*	154	31 (10)	123	2	0.25	75	32.421	66.737
*Ph. perniciosus* (PN)	41	6	35	3	0.04	75	8.632	75.369
*Ph. alexandri*	33	13	20	4	0.65	50	6.947	82.316
*Ph. riouxi*	31	10	21	5	0.47	50	6.526	88.842
*Ph. longicuspis* (LC)	29	0	29	6	–	50	6.105	94.947
*Se. fallax*	12	10	2	7	5.00	50	2.526	97.473
*Ph. papatasi*	6	2	4	8	0.50	50	1.263	98.736
*Se. dreyfussi*	2	0	2	9	–	25	0.421	99.157
*Ph. langeroni*	1	0	1	10	–	25	0.211	99.368
Damaged	3	0	3	–	–	50	0.632	–
Total	475	133 (12)	342	–	0.38	–	100	–

### Seasonal occurrence, diversity, and species richness of collected arthropods

**Figure 1:**
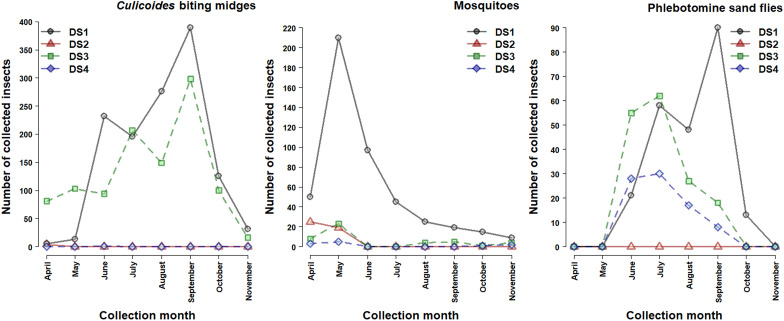
Monthly abundance of blood-sucking arthropods collected at the four dog shelters (DS).

**Figure 2:**
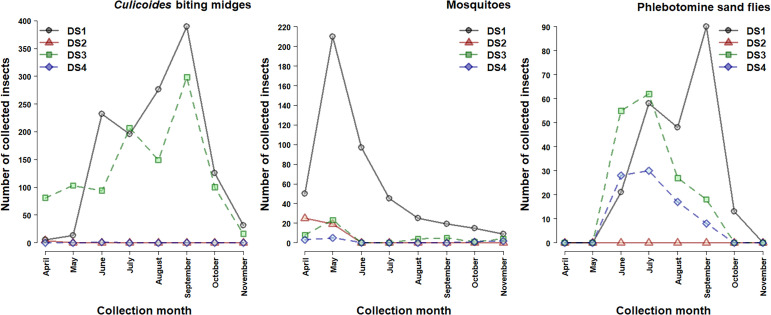
Seasonal variations of the most abundant *Culicoides* species trapped at DS1 and DS3.

The abundance of *Culicoides* populations, already active in April, grew progressively to reach a first peak in June in DS1 and in July at DS3. Then, the second peak occurred in September followed by a rapid population decrease in October/November (Fig. 1). Very few *Culicoides* were collected in DS2 and DS4. *Culicoides imicola* was the dominant species, with an abundance peak in September (Fig. 2) at both sites (42.90% of the total collection at DS1 and 71.0% at DS3), whereas the rank of other species variated between sites. *Culicoides paolae* was abundant at DS1 (29.00%), with an abundance peak in June and August, but rare at DS3 (< 0.50%). *Culicoides circumscriptus* was the third most abundant species at DS1 (12.20%), peaking in August, with a similar abundance to *C. newsteadi* (9.40 and 8.10%) at DS3, where the latter peaked in September (Fig. 2). The richness and diversity were maximum at DS1 (H’ = 1.57; S = 13) and the DS3 (H’ = 1.09; S = 13), while observed biodiversity was lowest at DS2 (H’ = 0.56; S = 2) and DS4 (H’ = 0.00; S = 1) (Table 4).

**Table 4:** Total number and species richness of *Culicoides* biting midges (Diptera: Ceratopogonidae) at the four dog shelters (DS) studied.

	DS1				DS2				DS3				DS4				Total			
Species	T	♀	♂	Rank	T	♀	♂	Rank	T	♀	♂	Rank	T	♀	♂	Rank	T	♀	♂	Rank
*C. imicola*	544	465	79	1	0	0	0	–	754	731	23	1	1	1	0	1	1,299	1,197	102	1
*C. paolae*	368	165	203	2	0	0	0	–	5	2	3	10	0	0	0	–	373	167	206	2
*C. circumscriptus*	155	40	115	3	0	0	0	–	84	63	21	3	0	0	0	–	239	103	136	3
*C. newsteadi*	32	18	14	6	3	3	0	1	99	99	0	2	0	0	0	–	134	120	14	4
*C. sahariensis*	41	10	31	4	0	0	0	–	10	4	6	7	0	0	0	–	51	14	37	5
*C. kingi*	27	2	25	8	0	0	0	–	23	18	5	5	0	0	0	–	50	20	30	6
*C. puncticollis*	35	19	16	5	0	0	0	–	7	7	0	8	0	0	0	–	42	26	16	7
*C. kurensis*	30	6	24	7	0	0	0	–	6	3	3	9	0	0	0	–	36	9	27	8
*C. saevus/langeroni*	4	2	2	11	0	0	0	–	31	22	9	4	0	0	0	–	35	24	11	9
*C. univittatus*	19	13	6	9	0	0	0	–	10	3	7	7	0	0	0	–	29	16	13	10
*C. longipennis*	0	0	0	13	0	0	0	–	12	10	2	6	0	0	0	–	12	10	2	11
*C. obsoletus/scoticus*	7	3	4	10	1	1	0	2	1	1	0	11	0	0	0	–	9	5	4	12
*C. submaritimus*	1	1	0	12	0	0	0	–	1	1	0	11	0	0	0	–	2	2	0	13
*C. clastrieri*	1	1	0	12	0	0	0	–	0	0	0	12	0	0	0	–	1	1	0	14
Damaged	4	4	0	–	0	0	0	–	5	5	0	–	0	0	0	–	9	9	0	–
Total	1,268	749	519	–	4	4	0	–	1,048	969	79	–	1	1	0	–	2,321	1,723	598	–
Species diversity	13				2				13				1				14			
ShannonWiener’s diversity	1.57				0.56				1.09				0				1.52			

**Figure 3:**
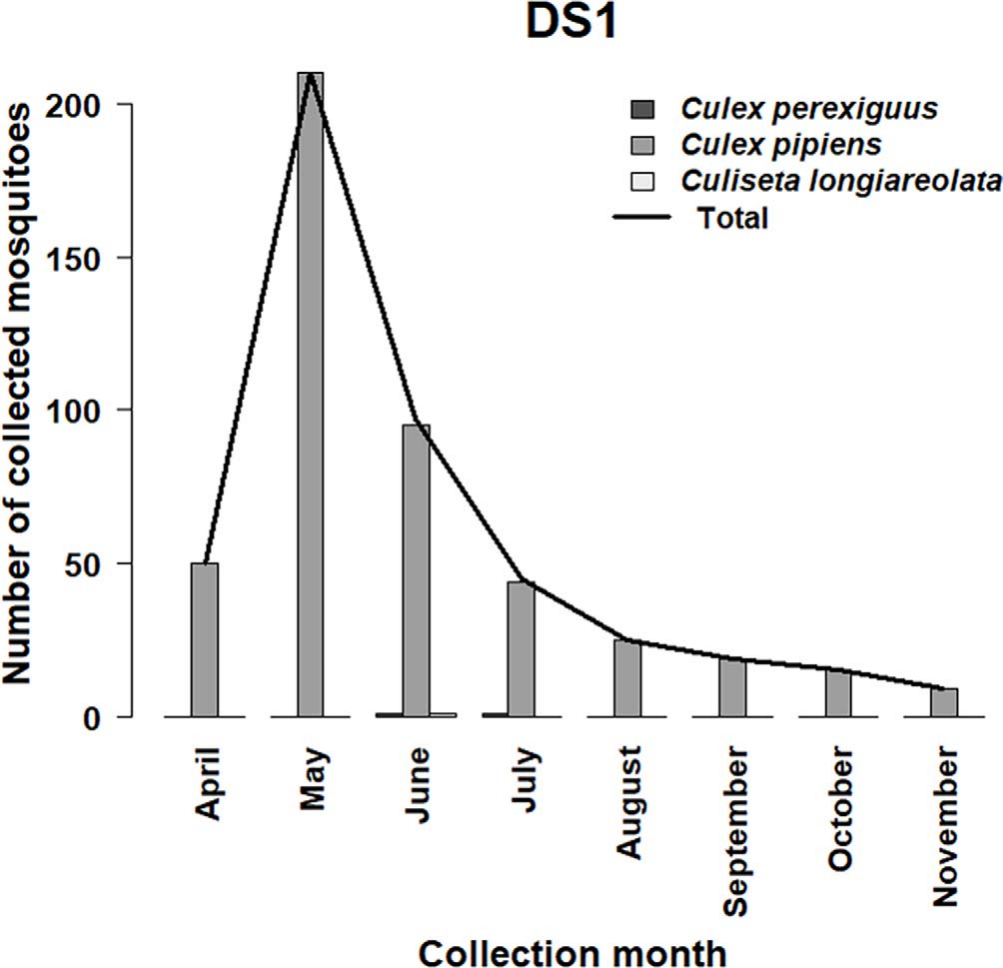
Seasonal variations of the most collected mosquito species caught at DS1.

For mosquitoes, the activity season extended from April to November (8 months) with a marked peak observed in May (Fig. 1). Except in DS1, where the majority of specimens were collected (82.40%), the mosquito fauna in the other DS remained very limited (17.60%). *Culex pipiens* s.l. was the most abundant species (96.84%), collected mainly at DS1, and presented a unimodal pattern (Fig. 3). The highest number of specimens was observed in May. In contrast, due to the scarcity of species in the other DS, the study of seasonality remains impossible, which explains the low values of the biodiversity parameters (Table 5).

**Table 5:** Total number and species diversity of mosquitoes (Diptera: Culicidae) at the four dog shelters (DS) studied.

	DS1				DS2				DS3				DS4				Total			
Species	T	♀	♂	Rank	T	♀	♂	Rank	T	♀	♂	Rank	T	♀	♂	Rank	T	♀	♂	Rank
*Cx. pipiens* s.l.	467	428	39	1	40	38	2	1	37	37	0	1	8	8	0	1	552	511	41	1
*Cs. longiareolata*	1	0	1	3	4	3	1	2	8	6	2	2	3	3	0	2	16	12	4	2
*Cx. perexiguus*	2	2	0	2	0	0	0	3	0	0	0	3	0	0	0	3	2	2	0	3
Total	470	430	40	–	44	41	3	–	45	43	2	–	11	11	0	–	570	525	45	–
Species diversity	3				2				2				2				3			
Shannon-Wiener’s diversity	0.04				0.30				0.47				0.59				0.15			

**Figure 4:**
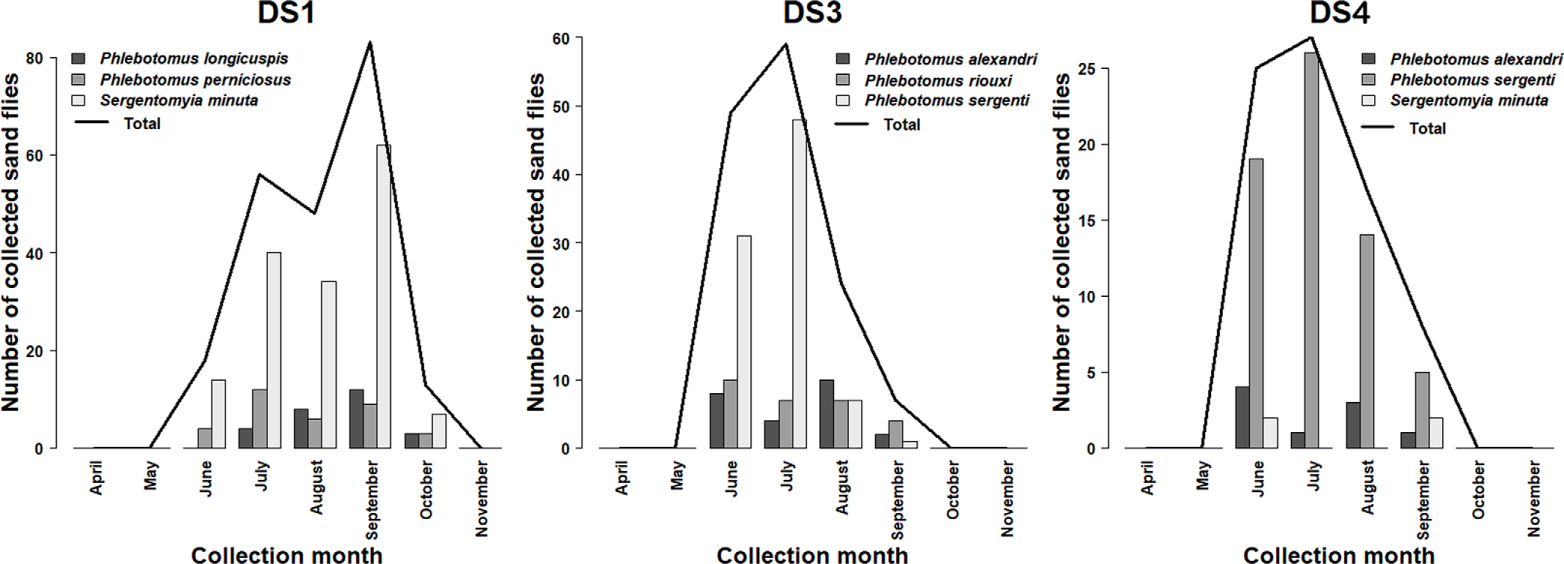
Seasonal variations of the most prevalent sand fly species collected at DS1, DS3, and DS4.

For the sand flies, the populations were active from June to October (Fig. 1) (5 months) at DS1, DS3, and DS4 with two peaks in July and September. On the contrary, no sand fly was collected in DS2. *Sergentomyia minuta* was the most abundant species (68.30%) followed by typical *Phlebotomus perniciosus* (PN) (14.80%), and typical *Ph. longicuspis* (LC) (11.80%) in DS1 (Fig. 4). The first two species displayed a bimodal trend with a first peak observed in July and the second most remarkable in September, while the third species showed a unimodal pattern of temporal distribution with a peak in September. *Phlebotomus sergenti* was the most abundant species at DS3 and DS4 (53.70% and 77.10%, respectively) with a peak in July followed by *Ph. riouxi* (17.30%) (abundance peak in June), and *Ph. alexandri* (14.80%) (abundance peak in June and August) at DS3 (Fig. 4). The abundance of other species remains very low at DS4 (Fig. 4). The biodiversity was greater at DS3 (H’ = 1.41; S = 8) than at the other DS (DS1 (H’ = 0.99; S = 7) and DS4 (H’ = 0.81; S = 6)) (Table 6).

**Table 6:** Total number and species diversity of phlebotomine sand flies (Diptera: Psychodidae) at the four dog shelters (DS) studied.

	DS1				DS2				DS3				DS4				Total			
Species	T	♀	♂	Rank	T	♀	♂	Rank	T	♀	♂	Rank	T	♀	♂	Rank	T	♀	♂	Rank
*Se. minuta*	157	55	102	1	0	0	0	–	2	2	0	7	4	4	0	3	163	61	102	1
*Ph. sergenti*	3	2	1	5	0	0	0	–	87	21	66	1	64	8	56	1	154	31	123	2
*Ph. perniciosus* (PN)	34	5	29	2	0	0	0	–	6	0	6	5	1	1	0	5	41	6	35	3
*Ph. alexandri*	0	0	0	–	0	0	0	–	24	10	14	3	9	3	6	2	33	13	20	4
*Ph. riouxi*	0	0	0	–	0	0	0	–	28	10	18	2	3	0	3	4	31	10	21	5
*Ph. longicuspis* (LC)	27	0	27	3	0	0	0	–	2	0	2	7	0	0	0	–	29	0	29	6
*Se. fallax*	4	3	1	4	0	0	0	–	8	7	1	4	0	0	0	–	12	10	2	7
*Ph. papatasi*	0	0	0	–	0	0	0	–	5	2	3	6	1	0	1	5	6	2	4	8
*Se. dreyfussi*	2	0	2	6	0	0	0	–	0	0	0	–	0	0	0	–	2	0	2	9
*Ph. langeroni*	1	0	1	7	0	0	0	–	0	0	0	–	0	0	0	–	1	0	1	10
Damaged	2	0	2	–	0	0	0	–	0	0	0	–	1	0	1	–	3	0	3	–
Total	230	65	165	–	0	0	0	–	162	52	110	–	83	16	67	–	475	133	342	–
Species diversity	7				0				8				6				10			
Shannon-Wiener’s diversity	0.99				0				1.41				0.81				1.69			

## Discussion

The entomological survey highlighted the composition, abundance, and diversity of blood-sucking arthropods collected at four dog shelters (DS) in central Morocco over an eight-month period from the beginning of April to the end of November 2022. The monitoring of these blood-sucking arthropods during 64 catch nights, allowed us to identify a total of 2,321 *Culicoides* biting midges (Diptera: Ceratopogonidae), 570 mosquitoes (Diptera: Culicidae), and 475 sand flies (Diptera: Psychodidae).

### Species composition

This study demonstrated the presence of at least fourteen *Culicoides* species at DS1 and DS3. The observations of González *et al.* [51] showed the same number of species collected in three dog and cat shelters located in northern Spain. *Culicoides* were absent in DS2 and DS4. This lack of observed specimens can be explained by the absence of the humid organic matter necessary for the breeding of biting midges in the vicinity of the traps. Furthermore, the regular use of volatile insecticides at DS2 could explain the general absence of arthropods in this shelter. Regarding DS4, the fact that it is located in a windy area limits the population of blood-sucking arthropods observed throughout the study period. *Culicoides imicola* was the most collected species in the present study, and is the most common species in Morocco and frequently collected in countries with a Mediterranean climate, especially on livestock farms [5, 15, 45, 55]. This abundance indicates the presence of moist clayey muds, which provide the specific larval sites required for the development of this species larvae [18]. Generally, *Culicoides imicola* is considered to be a heliophilous species resistant to desiccation, which explains its abundance in warmer regions [32]. The second most abundant species at DS1 was *Culicoides paolae* (29.0%), whereas at DS3, it was *C. newsteadi* (9.4%). The differences in abundance noted in these two study areas may be related to ecological parameters. The high abundance of *Culicoides paolae* at DS1 is associated with the presence of decaying prickly pear trees requiring clay-rich soil and high salinity, which provides a suitable environment for egg-laying by females [13, 74]. The first record of *Culicoides paolae* in Morocco was in 2019 [14]. This species remains among the most frequently encountered *Culicoides* species in several studies carried out in Tunisia [97], Italy [46], and Malta [50]. The observed abundance of *Culicoides newsteadi* in DS3 is connected to the presence of water-saturated organic matter, which constitutes the optimal breeding site for this species [4, 18]. *Culicoides circumscriptus* remained the third most frequently collected species in DS1 (12.2%) and DS3 (8.1%). The capacity of *Culicoides circumscriptus* to adapt to different habitats may explain its similar presence at DS1 and DS3 [102].

Regarding mosquitoes, *Culex pipiens* s.l. (96.84%) was the most dominant species in the current study, which corroborates the results of Filali Mouatassem *et al.* [44] and González *et al.* [51], where *Cx. pipiens* s.l. was the most abundant species in Morocco (89.92%) and Spain (76.80%). DS1 had the highest number of specimens collected compared to DS2, DS3, and DS4, which may be associated with the availability of an aquatic environment required for larval development [44, 72]. In the present study, low biodiversity was observed in terms of mosquito species compared to that observed in Spain (eight species; González *et al.* [51]), which is perhaps correlated to the presence of anthropogenic larval habitats (water containers, waste water) at the studied sites, where *Culex pipiens* s.l. is mainly found alone [2]. This species prefers water rich in organic matter, and its abundance is positively correlated to urbanization and agriculture optimizing its nuisance [2]. It should be noted that a few specimens of *Culiseta longiareolata* were collected during this study. These findings are consistent with what is found in dog and cat shelters in northern Spain [51]. It is known that these two species share the same breeding sites, and consequently the differences in their observed abundance seem more likely to be linked to competition in the same larval habitats [71].

Unlike the results of González *et al.* [51], which indicated a total absence of sand flies in dog and cat shelters in northern Spain, our study highlighted substantial diversity of these blood-sucking arthropods, which varied between DS1, DS3, and DS4 depending on biotic and abiotic factors [99]. The lack of sand fly species in DS2 is probably related to the regular treatment of all dogs in this shelter with collars containing 10% imidacloprid and 4.5% flumethrin. This is consistent with the results of a study carried out in a dog shelter in Italy, where the use of these collars had a reducing effect on the composition of the sand fly fauna [85]. Ten sand fly species were identified in the current study. In southern Italy, five species of sand fly were collected at a dog shelter [100]. In contrast, only two species were reported in another study carried out at a dog shelter in Lecce, Italy [76]. *Sergentomyia minuta* was the dominant species in our study, accounting for 34.31% of total catches in the three DS. Its greatest abundance (68.30%) was observed at DS1. This finding matches three previous studies in dog shelters in Italy [67, 76, 100], in which this species represented 92.70%, 46.46%, and 45.10% of all sand flies collected. This species is more frequently collected in dry, open areas and sandy soils in rural and urban settings [16, 34], similar to the conditions described at DS1 [59]. On the other hand, *Phlebotomus sergenti* was the most abundant species at DS3 (53.70%) and DS4 (77.10%), representing 32.42% of all specimens collected in the present study. These observations are in contrast with those of Latrofa *et al.* [67], Mendoza-Roldan *et al.* [76], and Tarallo *et al.* [100] who reported the absence of this species from dog shelters in southern Italy. This high abundance indicates their preference for the sandy-loam soils observed at these two DS [59]. Typical form of *Phlebotomus perniciosus* (PN) and *Ph. longicuspis* (LC) remain the second (14.80%) and third (11.80%) most collected species at DS1, respectively. *Phlebotomus perniciosus* is characterized by endo-exophilic behavior and is widely distributed in northern and south-western Morocco [52, 104], whereas *Ph. longicuspis* has an exophilic character with preference for sandy-loam soils [59, 104]. On the contrary, *Phlebotomus riouxi*, which remains a rare species in the Maghreb region and *Ph. alexandri*, which is very common in urban areas, were the most encountered species at DS3 [8, 30].

### Seasonal activity

Our findings are in line with those of González *et al.* [51], in northern Spain, who observed a marked peak of *Culicoides* in early summer and a decrease in their activity in autumn. These observations disagree with those of previous entomological studies in Morocco [13, 69], in which the most remarkable peaks occurred in spring and autumn.

Regarding the *Culicoides* species, *Culicoides imicola* showed a peak of activity in September in Rabat, which is in agreement with Bourquia *et al.* [15]. It is worth noting that there was a sudden decrease in abundance in July at DS1 and in August at DS3; this could be explained by the decrease of vegetation and forest cover during this period, which is unfavorable to *Culicoides imicola* [6, 37]. The population of this Afrotropical species is known to be affected by wind speed, which impacts their flight activity [6]. *Culicoides paolae* presented two peaks observed in June and August. This finding is in discordance with that documented in Andalusia (southern Spain) by Estrada *et al.* [42], who reported that September and October were the most abundant months for *Culicoides paolae*, which prefers the Mediterranean climate. Our study survey highlighted the abundance of *Culicoides newsteadi* during summer, which is contradictory with other reports in Morocco and Italy where *C. newsteadi* was more abundant in spring [15, 47]. Thus, this species is characterized by its ability to tolerate the highest minimum temperatures [4]. The higher number of *Culicoides circumscriptus* was observed in summer. This result is supported by Ortega *et al.* [82] during their study in southern Spain, indicating that this species reaches its peak of activity in summer, marked by highest maximum temperatures. Previous observations have reported this species on cattle farms close to coastal plains, and it has regularly been described in humid and sub-humid zones of Morocco [13, 46].

Regarding the seasonal pattern of mosquito populations, this study demonstrated a peak in abundance in late spring, which is in accordance with the findings of Marc *et al.* [72], where the peak of *Culex pipiens* s.l. activity in the province of Kénitra (Morocco) occurred in June. In contrast, the activity of mosquitoes studied in Spain showed a sinusoidal dynamic with a peak in abundance in early summer [51]. This can be explained by the impact of weather on mosquito population habitats in the study areas concerned. Several studies have found that high temperatures accompanied by a lack of precipitation in summer reduce aquatic breeding sites for *Culex pipiens* s.l., confirming that this species is affected by drought [20, 92]. In addition, early precipitation conditions contribute to their increased abundance, while the extension of their season is a consequence of late rainfall conditions [36].

Regarding the seasonal activity of sand flies, our findings align with those of Talbi *et al.* [99] in Aichoun (central Morocco) and Zarrouk *et al.* [104] in Tétouan city and Oued Laou village (northern Morocco), who reported the bimodal seasonal trends of sand fly populations with two remarkable peaks occurring in July and September. However, in contrast to these findings, other entomological investigations carried out in Morocco have shown that sand flies exhibit two significant peaks of abundance in June and August in Zlililgh (central Morocco), and in August and October in Taza (central-north Morocco) [53, 65]. These seasonal fluctuations could be associated with the climatic conditions that influence their life cycle [49, 104]. Their activity duration remains short, extending from June to October in the present study. This finding is in line with what is reported in other studies in northern and central Morocco indicating that the seasonal activity of sand flies begins in summer and ends in autumn [99, 104]. The activity of *Sergentomyia minuta* showed a bimodal cycle, as reported in Marrakech city (central Morocco) by Boussaa *et al.* [17] and it is well distributed in a semiarid climate [59]. On the other hand, *Phlebotomus sergenti* peaked in July in this study and another in Marrakech [17]. While in northern Morocco, this species experienced two peaks in abundance: the first in July and the second in September [104]. According to Kahim *et al.* [59], this species is distributed in several bioclimatic stages and its abundance is favored by a semiarid climate. In this study, *Phlebotomus perniciosus* (PN) displayed two peaks recorded in July and in September as stated in previous studies conducted in Spain and Morocco, which is abundant in humid to semi-arid climates [49, 52, 104]. *Phlebotomus longicuspis* (LC) which was sympatric with *Ph. perniciosus* (PN) in DS1 presented a unimodal seasonal distribution with peak activity in September. These results are in agreement with those of Guernaoui *et al.* [54] in southwest Morocco and it is well described in Saharan areas [59]. *Phlebotomus riouxi* is frequently collected in arid and hyper arid climates, while the abundance of *Ph. alexandri* increases in subhumid to arid zones [8, 30].

### Vector role

Regarding their role in disease transmission, *Culicoides* are potential vectors of Bluetongue Virus (BTV) in livestock, which is endemic in Morocco [33]. The peak abundance of *Culicoides* observed in our study coincides with very high BTV infection rates [70]. Interestingly, of the 1,723 females collected in the current study, 34 (2.00%) were blood fed (27 *Culicoides imicola*, 3 *C. newsteadi*, 2 *C. saevus/langeroni*, 1 *C. circumscriptus*, and 1 *C. longipennis*). In contrast, blood fed females were absent in the collections of *Culicoides* found by González *et al.* [51] in northern Spain. Slama *et al.* [97] confirmed the canine origin of the blood meal in a female *Culicoides imicola*, which can feed on livestock, birds, and humans. Furthermore, many *Culicoides* species including *Culicoides obsoletus*, *C. punctatus*, and *C. pulicaris* are known to feed on dogs in Siberia [78]. In contrast, analysis of blood meals from 400 females collected in Israel and Zimbabwe did not suspect the dog as the host species. Oura and El Harrak [87] reported the BTV infection of 21% of dogs in Morocco infected by the bites of *Culicoides*. According to Hanekom *et al.* [56], an infected dog suffers from anorexia, lethargy, dyspnea, and hypoxia, but remains unable to replicate the virus [86], which can be transmitted by direct contact or ingestion of infected meats from infected sheep [55]. With regard to African Horse Sickness Virus (AHSV), this is another *Orbivirus* that has been the subject of a few studies in dogs worldwide. Van Sittert *et al.* [103] reported the death of a dog in South Africa due to this virus, which never had access to horse meat, although Braverman and Chizov-Ginzburg [19] concluded that vector transmission in the case of AHSV in dogs remains accidental, as it cannot be a reservoir and its role in epidemiology is neglected. Of note, a recent study in Italy showed the presence of *Dirofilaria immitis* and *D. repens* DNA in *Culicoides paolae*, which are mosquito-borne nematodes [80].

*Culex pipiens* is the main vector of West Nile Virus (WNV), that has caused several epizootics in horses in Morocco [43]. According to El Rhaffouli *et al.* [41] and Assaid *et al.* [3], this emerging arbovirus (arthropod-borne virus) affecting humans is still circulating in Morocco. A few studies have demonstrated that dogs are highly exposed to WNV: the seroprevalence rate found in Morocco was 62% [38], whereas it was 30.2% and 8.1% in Libya [11] and France [66], respectively. The dog population remains an effective means of measuring the circulation and spread of the virus in a given area [11, 38, 66]. Consequently, it seems highly appropriate to set up a sentinel surveillance system at dog shelters in various parts of the kingdom, located in rural and peri-urban areas close to the human population, in order to obtain data on viral exposure and intervene at the appropriate time to overcome the damage associated with WNV on animal and human health, which is still endemic in North and West Africa [10]. *Culex pipiens* is also a vector of *Dirofilaria immitis* and *D. repens*, whose reservoir is the dog, and can be transmitted to humans [21, 24, 91]. In addition, human exposure to these filarial infections is favored by an endemic context, as is the case in the Mediterranean basin [77]. *Dirofilaria immitis* was more prevalent (14.5%) than *D. repens* (3%) in dogs tested in central and northern Tunisia [91]. However, *Dirofilaria repens* infection was more predominant in dogs from shelters located in southeastern Spain [25] and southern Romania [29]. As for Morocco, the status of these mosquito-borne nematodes is unclear, so regular screening of mosquitoes and dogs for *Dirofilaria* sp. is still highly recommended to update data and implement an appropriate control plan.

It is well known that *Sergentomyia minuta* is implicated in the transmission of *Leishmania tarentolae* (Subgenus: *Sauroleishmania*), which mainly infects geckoes but can also affect other reptiles, such as lizards [76]. The dog is exposed to *Leishmania tarentolae* without being a competent reservoir of the infection [76]. The fact that this species feeds on humans highlights its opportunistic behavior, well-illustrated in another study carried out in Italy, in which human DNA was found in 16 blood engorged *Sergentomyia minuta* [1]. In addition, *Leishmania major* has been detected in *Sergentomyia minuta* collected in southern Tunisia, where cutaneous leishmaniasis is endemic [58], as well as being involved in the transmission of Toscana virus in France [28]. Its vectorial capacity is optimized by its abundance in a leishmaniosis endemic area, which corresponds to the location of DS1 in central Morocco, where there are foci of human visceral and cutaneous leishmaniasis [34]. In Morocco, *Leishmania tropica* is transmitted exclusively by *Phlebotomus sergenti*, which is responsible for human cutaneous leishmaniasis and is also involved in the canine visceral form [68, 104]. Therefore, it seems that DS3 and DS4 are suitable environments for the emergence of new zymodemes and the spread of the disease on a large scale [90]. On the other hand, *Phlebotomus perniciosus* and *Ph. longicuspis* are potential vectors of *Leishmania infantum* infection, which causes zoonotic visceral leishmaniasis and zoonotic cutaneous leishmaniasis in several Mediterranean countries, including Morocco [104]. At DS1, the simultaneous presence of dogs, which play an epidemiological role as reservoirs of this protozoan, and the plant litter necessary for the development of the larvae of these species maintains the life cycle of the parasite and increases the risk of transmission of the disease, which is a major public health problem [60]. *Phlebotomus riouxi* can be incriminated as a vector of *Leishmania tropica*, as reported by Tabbabi *et al.* [98] in southeast Tunisia. The anthropophilic behavior of *Phlebotomus alexandri*, which prefers the peri-urban environment to feed on humans, may explain its abundance at DS3, and it has been suspected that it is involved in the life cycle of *Leishmania infantum* [30], suggesting its coexistence with *L. tropica*, which may contribute to the spread of infections and the appearance of new foci.

In summary, this descriptive study focused on entomological investigations of the arthropod fauna at four dog shelters in central Morocco, which determined the diversity, abundance, and composition of *Culicoides* (Diptera: Ceratopogonidae), mosquitoes (Diptera: Culicidae), and sand flies (Diptera: Psychodidae), with the aim of discussing their vectorial roles and assessing the risk of these shelters as zoonotic diseases foci. The high abundance of several potential vector species increases the risk of the circulation of many vector-borne pathogens, including those of zoonotic interest in Morocco, namely West Nile virus, *Dirofilaria immitis*, and *D. repens* transmitted by *Culex pipiens*, *Leishmania infantum* transmitted by the bites of infected *Phlebotomus perniciosus* and *Ph. longicuspis*, and *L. tropica* spread by infected *Ph. sergenti*. As a result, regular monitoring of vector-borne pathogens in dog shelters as well as arthropod fauna is strongly needed, to obtain sufficient data to set up surveillance and control systems for the risks assessed.

## Appendix

### Protocol used for the preparation of RNAlater-like solution (1.5 Litres)

- In a beaker, combine: 935 mL MilliQ H_2_O, 40 mL of 0.5M EDTA, 25 mL of 1M sodium citrate trisodium salt dihydrate, and 700 g of ammonium sulfate.
- Stir on a hot plate stirrer on low heat until the ammonium sulfate is completely dissolved.
- Allow to cool, adjust the pH of the solution to pH 5.2 using 1M H_2_SO_4._
- Transfer to a screw top bottle and store at room temperature.

Final concentrations:

- 25 mM sodium citrate trisodium salt dihydrate
- 10 mM EDTA
- 70% (w/v) ammonium sulfate
- pH 5.2
